# Evaluation of the Van Herick Technique for Screening for Occludable Angles in an African Population

**DOI:** 10.5005/jp-journals-10008-1142

**Published:** 2013-05-09

**Authors:** Shibal Bhartiya, Tarek Shaarawy

**Affiliations:** Consultant, Department of Ophthalmology, Fortis Memorial Research Institute, Glaucoma Services, Gurgaon, Haryana, India; Chief, Department of Ophthalmology, University of Geneva, Glaucoma Sector, Geneva Switzerland

**Keywords:** Van Herick technique, Peripheral anterior chamber depth, Occludable angles, Gonioscopy.

## Abstract

The current gold standard for screening for angle closure and adopting universal approaches to prophylaxis is the assessment of the anterior chamber (AC) angle by gonioscopy, a technique that has substantial interobserver variability and relies on subjective assessment. Slit-lamp estimation of the peripheral anterior chamber depth (ACD) by the Van Herick technique is a noncontact approach for estimating angle width and various authors have commented on its sensitivity and specificity as a screening tool for identifying narrow angles as well as angle closure.This case series draws attention to the fact that as many as 28 out of 36 (77.78%) seemingly open angles on Van Herick test were found to be potentially occludable angles on gonioscopy.

Therefore, it may be concluded that gonioscopy is essential even in patients with deep peripheral ACs, before an occludable angle can be ruled out.

**How to cite this article:** Bhartiya S, Shaarawy T. Evaluation of the Van Herick Technique for Screening for Occludable Angles in an African Population. J Current Glau Prac 2013;7(2):88-90.

## INTRODUCTION

A major challenge in screening for angle closure and adopting universal approaches to prophylaxis is the assessment of the anterior chamber (AC) angle. The current reference standard is gonioscopy, a technique that has substantial interobserver variability and relies on subjective assessment.^1^

Slit-lamp estimation of the peripheral anterior chamber depth (ACD) by the Van Herick technique^2^ is a noncontact approach for estimating angle width and various authors have commented on its sensitivity and specificity as a screening tool for identifying narrow angles as well as angle closure. There are divergent opinions regarding the usefulness of peripheral anterior chamber depth (PACD) measurement.^1-9^

We herein evaluate the usefulness of the Van Herick technique in ruling out narrow angles in glaucoma patients in Southern Egypt.

## MATERIALS AND METHODS

A total of 36 consecutive glaucoma patients, with a Van Herick test showing ACD greater than half of the corneal thickness, attending the glaucoma clinic of Kom-ombo Hospital were recruited for this study. The study conformed to the declaration of Helsinki and good clinical practice, and an informed consent was taken from each of the participants before inclusion. As no procedures apart from a routine glaucoma work up were performed, an approval from the ethical committee was not sought. A Van Herick assessment of the ACD was performed on the slit lamp by a single observer (SB) in order to identify the study population, and gonioscopy was then performed by the same observer.

### Technique: Van Herick Technique of Estimation of PACD

The grading of limbal chamber depth was carried out at a slit lamp (Model 900 BM, Haag-Streit, Bern, Switzerland). The illumination column was offset from the axis of the microscope by 60°, objective magnification was set to 1.6×, and the brightest, narrowest possible vertical beam of light was directed at the temporal limbus, perpendicular to the ocular surface, and viewed from the nasal aspect. The beam was positioned at the most peripheral point of the cornea allowing a clear view of the AC and peripheral iris.

The ACD was then graded as a fraction of the thickness of the adjacent cornea in the following categories: Grade 1 < 1/4, grade 2 = 1/4, grade 3 = 1 * 4 - 1/2, and grade 4 >full thickness of the peripheral cornea.^2-9^

### Technique: Gonioscopy

Gonioscopy was performed at a low level of ambient illumination using a Goldmann 3-mirror lens at high magnification (1.6×) with the eye in the primary position of gaze. A 1 mm light beam was reduced to a narrow slit, and the vertical beam was offset horizontally for assessing superior and inferior angles and vertically for assessing nasal and temporal angles. Care was taken to avoid light falling on the pupil during gonioscopy. Oxybuprocaine was used as a corneal anesthetic. A 2% hypromellose solution was used as a coupling medium for the contact lens. The Scheie's grading scheme, which is based on the angle structures visible during the examination, was used. AC angles were classified as occludable or nonoccludable. An occludable angle was defined as one in which the posterior trabecular meshwork was visible for less than 90° of the angle circumference, with gaze in the primary position.

The results of only those with an AC Van Herick's grade of greater than equal to three were used for analysis. The percentage of patients with seemingly open angles on Van Herick test but having occludable angles on gonioscopy was calculated.

## RESULTS

The average age of patients recruited in this study was 44.46 ± 9.36 years (range: 23-65 years). Of the 36 patients, there were 24 males and 12 females. The ACD was estimated on the slit lamp, 16 patients had a Van Herick grading of 3, while 20 had a grading of 4.

The results of gonioscopy in these patients are as shown in Table 1.

As many as 28 out of 36 (77.78%) seemingly open angles on Van Herick test were found to be potentially occludable angles on gonioscopy.

## DISCUSSION

Of the well-described ocular risk factors for primary angle closure glaucoma (PACG), including shallow AC, short axial length, small corneal diameter and thick crystalline lens, a shallow AC is the most consistent risk factor. ACD is an inheritable trait which is highly correlated with age, tends to be shallower in women and is influenced by ethnicity; tending to be deeper in Caucasians than Asians and shallowest in the Inuit Eskimos.^7,8^ There is, however, little epidemiological data on ACD in African population, with most hospital-based evaluations reporting an incidence of PACG ranging from 6.6 (Ghana)^10^ to 18% (Ethiopia).^11^ A population-based survey revealed the prevalence of PACG to be 0.5% (South Africa) of general population.^12^

The anatomic configuration of the AC angles in glaucomatous Nigerian adults, aged 30 years and above, revealed closed angles (Scheie grade 0 or 1) in 15.0% of cases compared to 1.6% of normals. A total of 38.8% of eyes with glaucoma were considered to have occludable angles compared to 10.4% of control eyes. Mean central anterior chamber depth (CACD) was shallower in glaucoma cases and females, decreasing with age in subjects with or without glaucoma. It was found to increase with increasing angle width, with just over half of subjects with closed angles having a mean CACD less than 2.5 mm.^13^

**Table 1:** **Table 1:** Van Herick grade 3 or greater and gonioscopy findings

*Angle on gonioscopy*		*Number of subjects*	
0		16	
1		8	
2		4	
3		4	
4		3	
5		1	

The measurement of PACD has been previously reported to be effective for the detection of PACG patients by Van Herick et al.^2,4,6,8^ A Van Herick ACD of greater than 3 is said to rule out angle closure. As per the original Herick et al report,^2^ ACD corresponding to grades 2-1 and 0 of their method can be regarded as carrying a risk of occlusion. Tajimi eye survey revealed that no eyes having a deep PACD, according to Van Herick's classification grades 3 and 4, were diagnosed with ACG.^5^

In our study, population of glaucoma patients from South Egypt, as many as 77.78% patients had potentially occludable angles in spite of having a deep peripheral AC contrary to popular belief.

This study is not without lacunae. It is not a population-based study, with a small sample size prone to bias as it comprises consecutive glaucoma patients in a referral center. The gonioscopy findings have also not been verified by objective quantification of angle width using any imaging technology.

Further population-based surveys to determine prevalence rates as well as an optimal screening protocol, for the African population are essential, where as many as 41 to 58% patients of glaucoma are blind in at least one eye.

## CONCLUSION

In spite of the obvious lacunae, the authors believe that the study is of relevance as a seemingly deep limbal AC does not imply that a gonioscopy may be avoided in these patients. As is evident from this case series, an ophthalmologist utilizing a gonioscope must make the final assessment even in patients with deep peripheral ACs, before an occludable angle can be ruled out.
